# Health research ethics in malaria vector trials in Africa

**DOI:** 10.1186/1475-2875-9-S3-S3

**Published:** 2010-12-13

**Authors:** Wen L Kilama

**Affiliations:** 1 African Malaria Network Trust, P O Box 33207 Dares Salaam, Tanzania

## Abstract

Malaria mosquito research in Africa as elsewhere is just over a century old. Early trials for development of mosquito control tools were driven by colonial enterprises and war efforts; they were, therefore, tested in military or colonial settings. The failure of those tools and environmental concerns, coupled with the desperate need for integrated malaria control strategies, has necessitated the development of new malaria mosquito control tools, which are to be tested on humans, their environment and mosquito habitats. Ethical concerns start with phase 2 trials, which pose limited ethical dilemmas. Phase 3 trials, which are undertaken on vulnerable civilian populations, pose ethical dilemmas ranging from individual to community concerns. It is argued that such trials must abide by established ethical principles especially safety, which is mainly enshrined in the principle of *non-maleficence*. As there is total lack of experience with many of the promising candidate tools (eg genetically modified mosquitoes, entomopathogenic fungi, and biocontrol agents), great caution must be exercised before they are introduced in the field. Since malaria vector trials, especially phase 3 are intrusive and in large populations, individual and community respect is mandatory, and must give great priority to community engagement. It is concluded that new tools must be safe, beneficial, efficacious, effective, and acceptable to large populations in the short and long-term, and that research benefits should be equitably distributed to all who bear the brunt of the research burdens. It is further concluded that individual and institutional capacity strengthening should be provided, in order to undertake essential research, carry out scientific and ethical review, and establish competent regulatory frameworks.

## Background

Without any doubt, malaria, a mosquito-borne disease, is a leading cause of morbidity and mortality in Africa, where it causes an estimated 300 million cases per annum, and around one million deaths, mainly of young children [1]. For sub-Saharan Africa (SSA), malaria is a cause as well as a consequence of poverty, costing SSA over US$12 billion per annum [2]. Overreliance on using only anti-malarial drugs has over the years led to successive resistance and subsequent loss of first-line drugs. Mosquito vector control is, therefore, needed in integrated malaria control, prevention and elimination, without which malaria parasite resistance to anti-malarial drugs would continue unabated [3].

Since existing vector control tools are turning blunt, and are few, biomedical research is being intensified in order to develop new mosquito vector control tools, which must be tested on humans, in the human environment, or on fabrics used by humans. Such interactions with humans at the individual, community and country levels and with the environment are likely to lead to ethical, safety, legal, regulatory, cultural, social and other concerns. There is also concern that entomology researchers do not usually consider ethical aspects of their research, while there is anecdotal evidence of their limited participation in ongoing training in health research ethics in Africa. This paper will focus on ethical concerns, with occasional allusions to some of the other concerns, especially safety. The paper should be of interest to researchers, sponsors and reviewers of mosquito research in Africa and beyond.

Health research ethics is mainly guided by four broad principles, namely, respect for a person’s autonomy, beneficience, non-maleficence and justice. For a concise description of these terms the interested reader should refer to the Declaration of Helsinki [4], and introductions to the guidance documents of the Council for International Organizations of Medical Sciences [5,6]; these guidance documents will be frequently referred to in this paper. For a more detailed discourse from an African perspective, readers may consult the paper by Tangwa [7].

Although human attempts at reducing mosquito bites are as old as human history, mosquito control strategies derived from research findings is only about a century old. In Africa, mosquito control tools were initially mainly developed during the early colonial period to safeguard colonized urban areas, mines and agricultural estates. Intensive efforts were invested in the research and development of malaria control tools during World War II, which resulted in the production and introduction of chloroquine and DDT [8], that later became the leading anti-malarial arsenal in the global malaria eradication programme of the 1950s and 1960s. It is also claimed [9] that insecticide-treated nets (ITNs) were initially developed for military use. Consequently, trials in the development of these insecticidal chemicals were initially undertaken in military facilities, on military personnel and later deployed on colonial agricultural estates, mines, and urban areas, which were the major beneficiaries. It is only recently that insecticidal products, particularly ITNs, have been specifically developed for majority civilian use in African populations.

This demands a paradigm shift in the process of developing products for malaria mosquito control. Whereas previously products were intended to protect individual short-term travelers, such as military personnel, colonial administrators, itinerant traders and tourists, the involvement of large, vulnerable, resident, civilian populations sets new demands in product development. In malaria mosquito control trials to be undertaken in Africa, the investigators, sponsors and reviewers of trial protocols must, therefore, be mindful of individual, community and environmental protections; equitable access to and sharing of benefits from research; and, given African realities, they should also address capacity strengthening not only of researchers, as is the case in clinical trials, but also of trial communities and institutions to be involved in the trials.

Unlike clinical trials, vector control trials will of necessity involve population-wide testing, which may infringe on individual freedoms, but can result in significant benefits for populations.

This paper will attempt to illustrate the ethical and practical concerns with available and candidate vector control strategies, and make attempt at addressing them.

## Epidemiological studies

Entomological studies are a crucially important component of malaria epidemiological studies. Mosquitoes must be collected in very early studies, so as to incriminate species responsible for malaria transmission, their feeding, biting, resting and other habits, and their susceptibility to prospective control measures. The collection of female mosquitoes, which is often undertaken in such studies, can be dangerous to mosquito collectors, who are sometimes referred to as mosquito bait, and some entomologists still prefer man-landing catches [10]. Quite often mosquito collectors are poor people, who undertake even the most demeaning tasks to scratch a living; they may, therefore, be classified as vulnerable research participants. In such situations, research staff are perpetrators of harms on fellow researchers. Surely mosquito collectors, who constitute an essential component of the research team must be protected; as humans, their autonomy must be respected. Indeed, their informed consent must be obtained before a study starts, and mechanisms must be put in place to ensure that the collectors understand and continue to understand the hazards involved in their participation.

Since in most cases mosquito collectors are employees of the research team, and are paid wages, some researchers argue against the requirement for informed consent. In all probability, the collectors got enrolled against their autonomy; because of poverty they cannot make a free choice. Moreover, it is very rare that senior researchers will offer themselves to undertake this or similar tasks, knowing the inherent harms relating to mosquito bites and the probability of transmitting malaria, filariae, and arboviruses, that are often endemic in study communities. Such involvement falls under the principle of *non-maleficence* (do no harm). In some situations, employment contracts create binding bondages, by requiring some employees to enter employment contracts as mosquito collectors. This author is of the opinion that even in such contracts informed consent by mosquito collectors be required, and should be periodically renewed. Information to be provided to a mosquito collector before s/he participates in the collection should include that contained in CIOMS Guideline 5 [6], more specifically:

• why s/he is considered suitable for the role to be played, and that participation is voluntary;

• the purpose, the procedures, duration, the recompense to be provided, foreseeable risks, pain, discomfort or inconvenience of the research;

• that the individual is free to refuse to participate and will be free to withdraw from the research at any time without penalty or loss of benefits to which s/he would otherwise be entitled;

• the expected benefits to the individual participant and the community;

• the sponsors of the research, the institutional affiliations of the investigators, and their funding;

• the extent of the investigator’s responsibility to provide services to the collector ;

• that treatment will be provided free of charge for research related conditions including infections from mosquito bites; and

• that an ethical review committee has approved or cleared the research protocol.

Furthermore, all out efforts should be made to use much safer mosquito collection methods, including light traps, double net traps, and animal bait.

## Existing vector control tools

Existing vector control tools include insecticidal chemicals (eg organochlorides, pyrethroids, organophosphates, and carbamates), biocontrol agents and environmental modification strategies. Because all these pose various risks to humans, and the mosquito habitats, from an ethical perspective, there is need for specific protections and restrictions. As a consequence, the safety of research participants, whether individuals or communities, and that of the environment must be given foremost attention. In the latter regard, the DOH under paragraph 13 requires that “Appropriate caution must be exercised in the conduct of medical research that may harm the environment”. For DDT, for example, there are many claims of toxicity to birds, carcinogenicity, and being anti-androgenic [11]. Exhaustive literature on both sides of the debate has been provided [12]. DDT supporters, however, vehemently deny the claims; they point to faulty study designs and interpretation, reasoning that even if DDT is dangerous, the gains made in saving so many human lives by far outweigh the harms [13-16] These debates led to the adoption of the Stockholm Convention on Persistent Organic Pollutants [17], under which DDT was not banned if used in accordance with WHO procedures. But later debates unleashed the precautionary principle [18], which puts the onus on those proposing the action to prove that the action is harmless. A Lancet editorial [19] appealed for “caution with the precautionary principle”; the latest WHO [20] position was adopted in the light of the precautionary principle.

Moreover in some trials consideration must be given to such aspects as dermal toxicity, corrosiveness to fabrics, and effects on treated surfaces. Indeed the “safe use of pesticides” has for decades been a WHO concern, especially as an occupational health hazard [21].

The adoption of a drug or vaccine for disease management is invariably based on the efficacy of the candidate product, which must be monitored to ensure continued efficacy. Indeed the DOH [4] under para 7 states that ” even the best current interventions must be evaluated continually through research for their safety, effectiveness, efficiency, acceptability and quality.” Similarly, with mosquito control tools, especially so with insecticides, efficacy must be established before the insecticide is adopted for use [22], and must be monitored regularly, particularly given the frequent threat of insecticide resistance. Monitoring must, therefore, include not only safety and efficacy but also continued assessment of effectiveness, efficiency, acceptability and quality. Failure to do so would be unethical as it would expose humans to poisonous or inefficient, unacceptable, and low quality products whose value in operational programmes is questionable.

The World Health Organization [23] has in place the WHO Pesticide Evaluation Scheme (WHOPES), which runs a four-phase evaluation and testing scheme, so as to establish the safety, efficacy and operational acceptability of insecticides intended for use in public health, and develops specifications for quality control and international trade. In particular, phase 1 tests for efficacy and persistency of technical products or their formulations using laboratory-bred arthropods; phase 2 studies natural mosquito populations in the field, on a small scale under well controlled conditions, and determining likely harmful effects upon operators; products passing phase 2 testing may get interim registration. Phase 3 assesses product efficacy under medium or large-scale field trials, including epidemiological evaluation; products passing phase 3 testing get full WHOPES registration. Phase 4, on the other hand, establishes specifications for the technical product [22]. Some African research institutions, including the Amani Research Centre and KCM College in Tanzania, CREC in Benin, Kow Valley in Burkina Faso, Pitoa in Nortern Cameroon, Akodessewa in Togo, Malanville in North Benin, participate as WHOPES testing sites for phase 2 and 3 trials.

In malaria control programmes, as exemplified in the development of ITN strategies, entomological efficacy must be followed by determining efficacy, for example, on child morbidity and mortality. During the development of ITN technology, some studies even determined parasitological efficacy, whereby attempts were made at determining reductions in parasite densities and fever [24]. Indeed in malaria endemic settings, where poverty is often rampant, feasibility, cost effectiveness, sustainability, durability and impact of the tested strategy must also be determined. From an ethical standpoint, when studies are over, under DOH 2008 para 33 the participants “ are entitled to … share any benefits that result from it, for example, access to interventions identified as beneficial in the study or other appropriate care or benefit”. This requirement is also covered under guideline 10 of CIOMS 2008, which states in part that “any intervention or product developed, or knowledge generated, will be made reasonably available for the benefit of that population or community.” In the body of CIOMS guideline 10, it is further stipulated that “if an investigational intervention has been shown to be beneficial, the sponsor should continue to provide it to the subjects after the conclusion of the study and pending its approval by a drug regulatory authority, when relevant”. It may be queried as to how long the sponsor’s obligation to provide the product should last and when it should end, or whether it is open ended. Besides, unlike in clinical trials, which are limited to individual research participants, entomological intervention trials in phase 3 may be widespread and providing a successful product to the entire study population may, therefore, be deemed impractical. Furthermore, a successful trial may demand building a new factory for manufacturing the trial product, when due to endemic poverty the market may be extremely weak, and the product unaffordable.

## Candidate vector intervention tools

The dawn, intensification and threat of the spread of insecticide resistance and environmental concerns have encouraged efforts in the discovery, and development of new mosquito vector control tools. These for example include new synthetic insecticides [25], entomopathogenic fungi [26], mosquito behavioural alterations [27], genetic control and genetic engineering. The latter is appropriately covered by Yeya Toure [28] in a paper in this issue. For an overall perspective of the field, the reader is referred to Takken and Knols [29]. These prospective vector control tools pose new challenges to national and international regulatory frameworks before their approvals for adoption in public health programs.

## Challenges and ethical implications in vector control trials

Phase 1 vector control research is entirely laboratory based and does not usually pose substantial ethical challenges. There are however ethical concerns where mosquitoes are fed on humans, or on laboratory animals. The ethics of the use of laboratory animals in African settings has been recently reviewed [30]. Ethical dilemmas in phase 2 testing relate mainly to operators who are exposed to the product under development. These operators must be adequately protected. In phase 3 trials, test products are applied under medium or large field scale including epidemiological evaluation, aiming at protecting entire communities, which in some trials can be in thousands or tens of thousands; there are inherent complex ethical challenges which may be extensive depending on the trial design.

As a consequence, in phase 3 vector control trials, individual informed consent cannot suffice; the research team is duty bound to obtain household, community, ward, or district-wide consent, depending on the extent of the spread of the intended intervention trial. The consent process in such studies is mind-boggling. Surely, community assent as currently used for example in community engagement would not suffice, since in vector control trials, especially where live mosquitoes are released, or in trials of aerial spraying, the entire community population constitutes research participants *per se*. Faced with such unwieldy situations, it has been argued by some ethicists that alternative means be found, for example by having a referendum, plebiscites or other democratic means, as a supplement to informed consent. The applicability of such approaches in community-wide malaria vector control trials in African settings are however doubtful.

It has been argued [31-36] that some candidate vector control technologies, in particular genetically modified mosquitoes, raise questions relating to the likely transfer of transgenes to non-target organisms, disturbing the culicine habitat, and the competitiveness of transgenic mosquitoes, thus again pleading for caution with these new technologies.

Some of the above challenges have been addressed by, for example, the introduction of contained (caged) field trials. The pertinent Scientific Working Group [37] has recommended:

• instituting security and safety oversight, through monitoring, biosafety, and regulatory organs.

• independent external audit;

• developing and using SOPs for safe keeping and transport,

• observing international covenants and protocols,

• observing national, regional, state or provincial, country or municipal, or institutional regulations,

It is, however, doubtful whether such regulations exist in prospective African countries intending to participate in such trials, and whether they are capable of enforcing them. Such regulations would for example cover:

• Release of organisms in the environment,

• General biosafety requirements,

• Research ethics and human participant requirements,

• Environmental health regulations,

• Occupational safety,

• Transportation regulations,

• Quarantine regulations, and regulations by funder and/or sponsor,

It is most probable that most African countries lack such regulations, and where they exist the regulatory bodies are weak and cannot, therefore, appropriately enforce them. Furthermore, at research institutional level there is insufficient strength to, for example, clear importation of live organisms, undertake scientific or ethical review [38], carry out monitoring or oversight. Consideration should, therefore, be given to initiation of supranational review mechanisms as is currently done under EDCTP for drug and vaccine trials in Africa[39]. The capacity strengthening of such regulatory frameworks must be given high priority.

## Case studies in malaria mosquito trials and control

**Figure 1:**
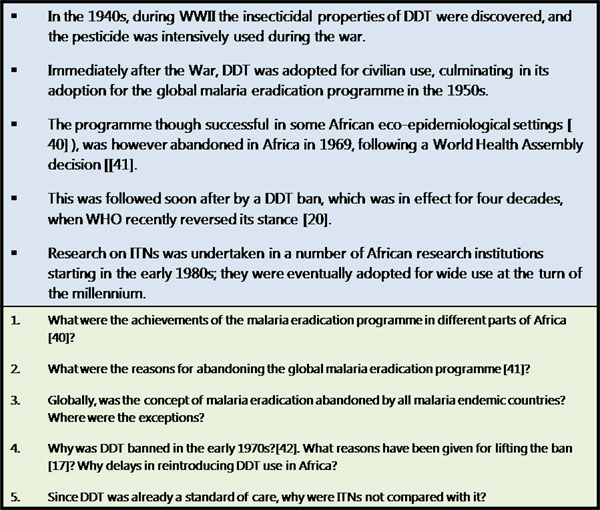
DDT Case Study: Chronological Events

**Figure 2:**
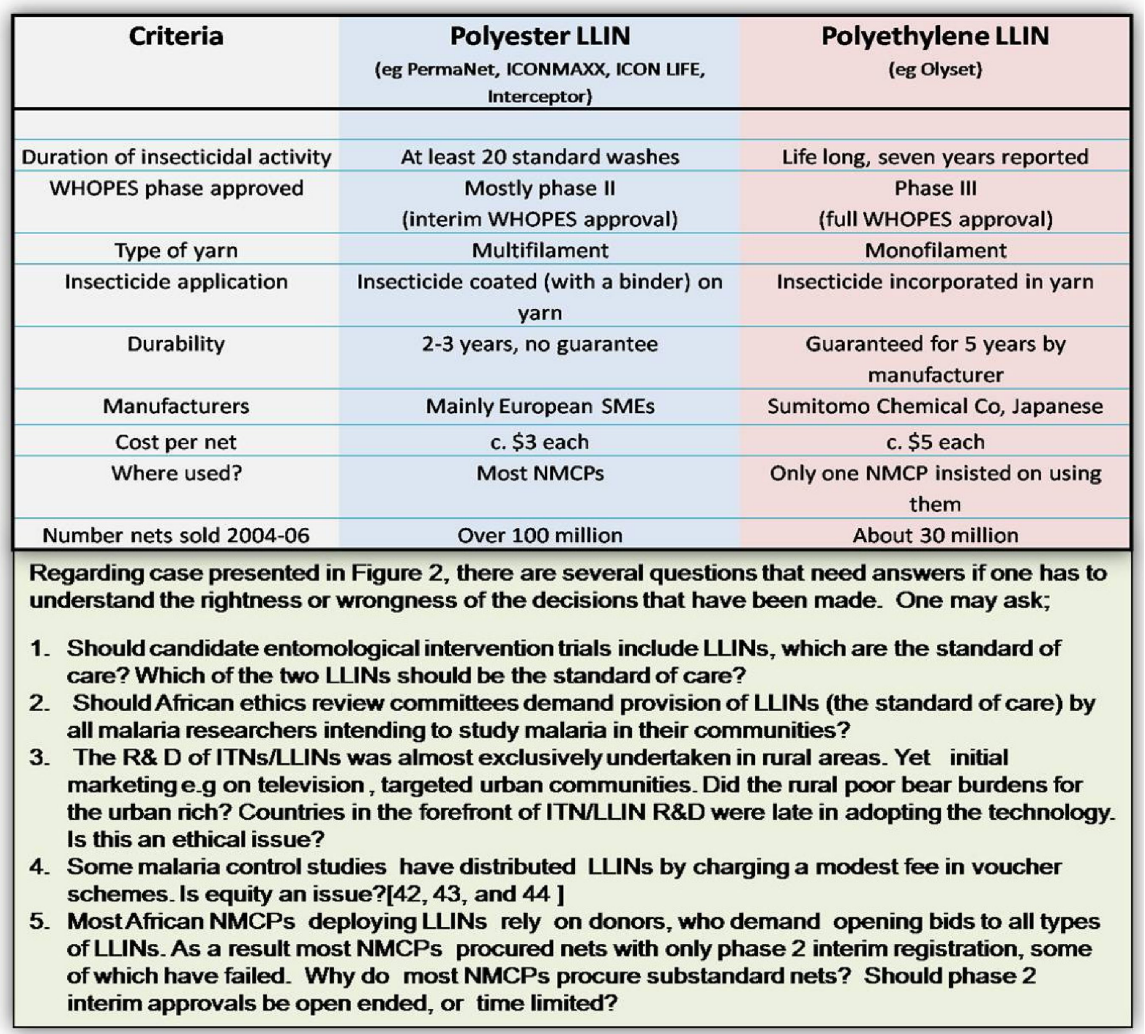
Comparison of two existing types of LLINs

Currently, insecticide-treated nets (ITNs) and long-lasting insecticidal nets (LLIN) are the mainstay of malaria control across SSA. In some areas indoor residual spraying is being implemented. LLIN deployment in community-wide control programmes has resulted in drastic malaria reductions, as has happened in Zanzibar, Eritrea, Madagascar, Rwanda, Sao Tome and Principe, and Zambia [1]. These phenomenal successes were preceded by a number of ethical issues during product development, as illustrated in the following case studies. (Fig.1 and 2).

## Community engagement

Community engagement has recently become popular in clinical trials and in epidemiological studies [45,46]. It is now commonly agreed that before a community-based study starts prospective study communities should be provided with clear non-technical descriptions of the intended study, including the thinking behind the study, its objectives, and the activities to be undertaken, aimed at obtaining community acceptance of the study. Since entomological trials are by their very nature community-based, and may be intrusive, community engagement should play a much greater role.

The acceptance of a new technology or for that matter accepting to participate in a study is not straight forward, especially with entomological trials, which frequently arouse suspicion and rumor-mongering [47-49]. In order to elicit community acceptance, it will be necessary to use various approaches, including drama, video, animation, and road shows. The study must be explained clearly, including its rationale and justification, any likely risks, their likely magnitude, and how to handle them. It is absolutely essential that the study team have an inbuilt mechanism to get feedback. Since informed consent is a process, it should not be rushed through; likely local concerns must be addressed adequately.

It is advisable that the researchers know study communities well; in this regard collaboration with social scientists, especially sociologists should be given high priority. Study teams should aim at building trust with study communities, to secure and sustain community endorsement and consent, establish means of communicating with communities, and give communities a sense of project ownership. Tindana[45], working in Ghana, has described the Navrongo model of community engagement, which starts with community entry, then holding community *Durbars*, followed by compound and household meetings, all leading to individual consent. Such procedures would equally be quite appropriate for other communities of SSA. What is abundantly clear is that in community wide studies the need to respect local behavior and etiquette is absolutely necessary. In entomological intervention studies the process would be more complex, and indeed it is not uncommon in such trials to have house entry refusals or research interventions confused with vector control interventions, an equivalent of therapeutic misconception in clinical trials. House entry refusals are not uncommon, especially with affluent households, where some may claim “not in my backyard”. Some successful projects have involved community advisory boards or community-owned resource persons [50]. But whatever the case, sustaining consent and rumor management are often difficult. During the study, all efforts should be made to minimize having non-consenting individuals, as they might wreck the entire project, since the entire study population constitutes a cohort. Indeed obtaining and sustaining informed consent throughout study communities, which would necessarily involve thousands of participants would be unwieldy. Even where consent is obtained, it would be extremely difficult, if not impossible to ensure that the consent obtained in such large trials is genuine, persists throughout the study population and is sustainable.

## Balancing benefits, harms and risks

Vector control strategies are a mixed bag in that they all contain potential benefits, harms, and risks. It is upon all stakeholders, encompassing researchers, sponsors, and the scientific and ethics review committees to make sure that in intended vector control trials there is a balancing of potential benefits, risks and harms, which in this case must also consider the community and the environment. Study designs must be examined to make sure that prospective intervention trials are at least as advantageous to the community and the environment, in the light of potential harms and benefits. Contingency plans must always be put in place for mitigating such “adverse events”, such as escape of GM mosquitoes, which may have increased vectorial capacity or vector competence for non-target pathogens, increase in mosquito fitness or longevity, changed blood feeding habits, decreased insecticide susceptibility, and horizontal gene transfer. It was these types of concerns that led to the development of contained field trial cages that are capable of minimizing potential escape of live mosquitoes, entomopathogenic fungi, and other exotic organisms. Even with such facilities security and safety oversight must be instituted, including their monitoring, inspection by regulatory organs, and independent external audits [37].

## Strengthening capacity for scientific, ethical and regulatory review

Before undertaking entomological studies, particularly those involving humans, intended study protocols must be examined by competent review committees for their scientific merit and ethical acceptability. In most African research institutions, scientific review precedes ethical review. This approach is mainly based on the principle that if the science is faulty, it may expose participants to risk or inconvenience to no good purpose and, therefore, the study cannot be ethical. Subsequently, it should be upon the scientific review committee to determine whether a cleared protocol should undergo ethical review. In case an entomological study claims that it is exempt from review, the research protocol should contain a statement to justify the claim. Nevertheless, such a protocol should receive competent administrative clearance that it is indeed exempt from review.

Such committees must be independent of the research team, be competent, and multidisciplinary. Since in most institutions medical entomologists may not be members of the ethics review committee, they should at least be co-opted on ad-hoc basis as need arises.

According to CIOMS [6] Guideline 20 “Many countries lack the capacity to assess or ensure the scientific quality or ethical acceptability of epidemiological research.” This dictum is very pertinent to malaria entomological research, since senior malaria entomologists are few and may not be members of either ethics or scientific review committees.

Besides capacity for scientific and ethical review of clinical and epidemiological research, countries and institutions should also have in place capacity for regulatory review of entomological protocols, providing monitoring and oversight of vector intervention trials, which would contribute immensely to the facilitation of national registration of new entomological intervention tools. In Tanzania for example the Tanzania Food and Drug Authority [51] does not undertake entomological review or oversight; this falls under Tanzania’s Tropical Pesticides Research Institute (TPRI)[52]. Malaria mosquito control is a component of public health, a very broad field encompassing many other fields. Unfortunately, health research ethics focuses attention on individual human protection, and as far as this author is aware, there are no ethical guidelines for public health research, including that on malaria mosquito trials. The development of such guidelines is long overdue, and should be given the priority it rightly deserves.

Fortunately, the WHO Special Programme for Research and Training in Tropical Diseases (WHO/TDR) is funding a project, designated MosqGuide, which is developing the needed guidance for genetically modified (GM) mosquitoes intended specifically to control malaria and dengue vectors. The guidance will support disease endemic countries and other stakeholders in considering the safety and legal/regulatory aspects, as well as ethical, cultural and social issues, of such deployment [53].

## Conclusions

Although malaria mosquito research in Africa is over a century old, it has hardly paid attention to ethical concerns, although the development of mosquito control tools must be tested on humans, on their fabrics, their dwellings, or on the environment, and may cause harm. In order to face the many ethical and practical challenges inherent in the research and development of new malaria mosquito control tools, it is paramount before studies start to provide comprehensive institutional capacity strengthening based on entomological holistic needs assessments. It should include strengthening ethical and regulatory review, developing ethical guidelines for public health intervention trials, which should include entomological aspects, while ensuring scientific and ethical review of entomological studies, co-opting entomological expertise as is deemed necessary. At the same time institutional, national and international regulatory frameworks, including WHOPES should be strengthened. New mosquito control tools must be tested beyond safety and efficacy, to include also effectiveness, acceptability, access, biosafety, and risk-benefit considerations, not forgetting environmental and health impact assessment. While undertaking trials ensure involvement of majority of stakeholders, promote participatory research and community engagement, and minimize rumour mongering. Since mosquito control trials are community-wide, aim at obtaining and sustaining community consent even though it is much more demanding than in typical cases of individual consent. As there are many justice issues during trials, and many more especially when trials are over, they must be adequately addressed.

## Glossary of Abbreviations

CIOMS: Council for International Organizations of Medical Sciences; DDT: Dichlorodiphenyltrichloroethane; DOH: World Medical Association Declaration of Helsinki; EDCTP: European Developing Countries Clinical Trials Partnership; GM: Genetically Modified ; ITN: Insecticide-Treated Net; LLIN: Long-Lasting Insecticidal Net; NMCP: National Malaria Control Programme; R&D: Research and Development; SME: Small to Medium Enterprise; SOPs: Standard Operating Procedures; WHO: World Health Organization; WHOPES: WHO Pesticide Evaluation Scheme; WW II: World War II

## Competing interests

The author declares that he has no competing interests.
